# Supplementary material for: Double-filtered leukoreduction as a method for risk reduction of transfusion-associated graft-versus-host disease
Source: PLoS One. 2020 Mar 26;15(3):e0229724. doi: 10.1371/journal.pone.0229724 (PMC7098637; doi:10.1371/journal.pone.0229724)
Supplement: Attachment — Submitted filename: Response to Reviewers.docx [file pone.0229724.s002.docx]

Dear Editor and Reviewers.

This is the document for our response regarding the revised manuscript of the article ‘Double-filtered leukoreduction as a method for risk reduction of transfusion-associated graft-versus-host disease’. We thank you for the time dedicated to reviewing our work and hope that our revised manuscript would be more fit for publication.

Journal Requirements:

1. When submitting your revision, we need you to address these additional requirements.

Please ensure that your manuscript meets PLOS ONE's style requirements, including those for file naming. The PLOS ONE style templates can be found at

<http://www.journals.plos.org/plosone/s/file?id=wjVg/PLOSOne_formatting_sample_main_body.pdf> and <http://www.journals.plos.org/plosone/s/file?id=ba62/PLOSOne_formatting_sample_title_authors_affiliations.pdf>

- The manuscript was ensured to meed PLOS ONE’s style requirements.

2. To comply with PLOS ONE submissions requirements, please provide methods of sacrifice in the Methods section of your manuscript.

🡪 This was added in the methods section. All mice were euthanized after experimentation, at day 43 by CO_2_ gas.

3. As part of your revision, please complete and submit a copy of the ARRIVE Guidelines checklist, a document that aims to improve experimental reporting and reproducibility of animal studies for purposes of post-publication data analysis and reproducibility: <https://www.nc3rs.org.uk/arrive-guidelines>. Please include your completed checklist as a Supporting Information file. Note that if your paper is accepted for publication, this checklist will be published as part of your article.

🡪 This was done. We have submitted a copy with our revised manuscript.

Reviewers' comments:

Comments to the Author

Reviewer #1: The authors present a study on an alternative approach (namely 2 subsequent leukoreduction by 2 distinct filtering techniques) to the current state of the art irradiation for the prevention of transfusion-associated graft-versus-host disease (TA-GVHD).

Major criticism:

1. The critical issue is to what extent does double filtration diminish the TA-GVHD triggering potential of the RBC preparation. In my opinion, this is insufficiently examined in this paper. This is attempted to be presented in the second half of Results section. About in vitro T cell expansion experiments 설명 보강, mere 2 sentences report. Similarly, critical circumstances are missing from the presentation of the in vivo mouse experiments of 12 lines. If one ultimately aims at an equivalency study with the gold standard technique one should have a more systematic approach to prove the hypothesis.

🡪 Thank you for raising this issue. We have also concerned about this in our initial manuscript. Further description of the limitation of the in vitro method, along with the meaning of our in vivo method was added. We have to admit that a systematic approach to fully prove equivalency to irradiation is impossible at the moment, and have revised our manuscript to focus on our results inidicating that a sufficient amount of cells are required to induce expansion (or GvHD, in the mouse experiment), and that our assays resulting in 1200-2500 cells per blood product is suggestive to be sufficiently below that. This itself is by no means sufficient to be an equivalency study. However, we hope that our results conclusion would shed some interest on the use of our proposed method in cases where irradiation is undesirable or impossible.

2. More detailed examination of quality changes following double filtration would also improve the paper. Quality parameter comparisons with those after irradiation are recommended.

🡪 This would be mainly summarized in Tables 1 and 2. More description was added to the manuscript on this issue. As this data would eventually be the main interest if one would apply double-filtration of RBC products, we hope that these tables would provide sufficient data to the readers.

3. The 2 terms: “leukocytes” and “lymphocytes” should not be used interchangeably.

🡪 This was also one of our concerns when submitting the initial manuscript. We have an explanation for this, and would like to clarify this issue. As leukoreduction filters target all leukocytes, in phrases where the main subject is leukoreduction itself, we used leukocytes. In the experimentation with mononuclear cells, the term PBMC or mononuclear cells were used. And when regarding the pathology of GvHD or other activities regarding T cells, the term lymphocytes were used. We have tried to be consistent on this throughout the manuscript, and did not use these as interchangeable terms. We feel that unifying these terms to a single one would be a less precise description.

4. The relationships of the tested double filtration procedure with that of the other alternative approach, namely the pathogen inactivation (reduction) should be discussed with relevant recent references (e.g. Kleinman, Transfusion 2018, Bahar, Arch Pathol Lab Med 2018). In the current text I did not find anything about this.

🡪 Thank you for bringing up this point. It would be poor practice not to include this in a study, and we are grateful that the reviewer brought up this point. We have to admit that pathogen reduction, with the up-to-date results on this method, can be a more promising method compared to filtration. We have discussed this issue in the discussion section.

5. Figure legends in general must be substantially extended with additional details e.g. case numbers in the respective subgroups, rational and method of subgrouping, statistics employed etc.

🡪 Figure legends were revised, and to further add detail in understading the context of each figure.

6. The quality of the abstract should be improved with more numbers characterizing the experiments performed and without company names.

🡪 Abstract was revised to be more explanatory of the results of this study.

Reviewer #2: The authors describe a technique by which 30 units of blood where subjected twice to filtration with filters provided by the Haemonetics Corporation. With these techniques, they could no longer detect leukocytes which they calculated to be less 1200 - 2500 cells. They substantiated their findings with in-vitro studies and in-vivo with immunodeficient mice. The experimental findings appear sound, however I recommend these changes:

1) The current standard world-wide is irradiation of blood products, since TA-GVHD is universally fatal, this cannot be changed easily and this needs to be re-iterated.

🡪 We agree with this point. This study itself cannot be the gounds to substitute irradiation, and we suggest this as a easy method to apply when irradiation is not desirable or impossible. This was also further discussed in the discussion section.

2) However, inactivation procedures have been implemented for platelets in the United States. This should be discussed in detail.

🡪 This was also an issue brought up by another reviewer. Thank you for commenting on this. It would be poor practice not to include this in a study, and we are grateful that the reviewer brought up this point. We have to admit that pathogen reduction, with the up-to-date results on this method, can be a more promising method compared to filtration. We have discussed this issue in the discussion section.

3) A second filtration obviously increases the cost of blood transfusion, therefore cost saving by not using an irradiator has to be balanced against the cost of using a second filter.

🡪 The cost of filtration was not a consideration when designing this study, as, said above, we do not suggest this as a substitute to irradiation, but more of an alternative method when irradiation is not desirable or not possible. This was further discussed in the discussion section. If double-filtration somehow gets further attention in the transfusion society, cost-productiveness would eventually have to be evaluated; however, as clinical trials on the efficacy of this would not be possible (due to too low prevalence and ethical issues). We feel that suggesting this as a method of last resort when irradiation cannot be done would be more fit.
